# Boronic Acid Functionalized Nanosilica for Binding Guest Molecules

**DOI:** 10.1021/acsanm.1c00005

**Published:** 2021-02-19

**Authors:** Xiaoting Xue, Haiyue Gong, Hongwei Zheng, Lei Ye

**Affiliations:** Division of Pure and Applied Biochemistry, Department of Chemistry, Lund University, Box 124, 22100 Lund, Sweden

**Keywords:** dendritic fibrous nanosilica, boronic acid, copolymer brush, Alizarin Red S, nicotinamide adenine dinucleotide, size exclusion

## Abstract

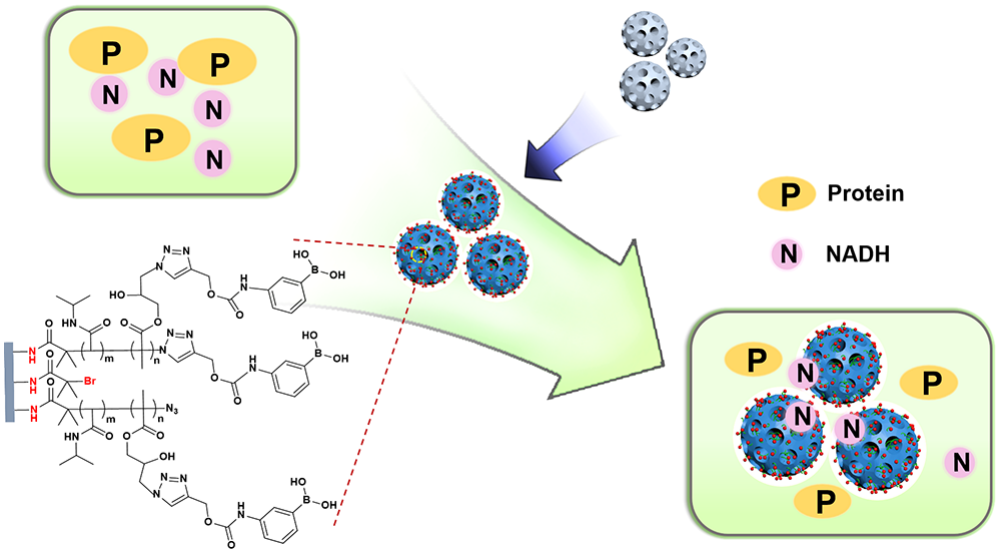

Dendritic fibrous nanosilica (DFNS) has very high surface area and well-defined nanochannels; therefore, it is very useful as supporting material for numerous applications including catalysis, sensing, and bioseparation. Due to the highly restricted space, addition of molecular ligands to DFNS is very challenging. This work studies how ligand conjugation in nanoscale pores in DFNS can be achieved through copper-catalyzed click reaction, using an optional, in situ synthesized, temperature-responsive polymer intermediate. A clickable boronic acid is used as a model to investigate the ligand immobilization and the molecular binding characteristics of the functionalized DFNS. The morphology, composition, nanoscale pores, and specific surface area of the boronic acid functionalized nanosilica were characterized by electron microscopy, thermogravimetric and elemental analysis, Fourier transform infrared spectroscopy, and nitrogen adsorption–desorption measurements. The numbers of boronic acid molecules on the modified DFNS with and without the polymer were determined to be 0.08 and 0.68 mmol of ligand/g of DFNS, respectively. We also studied the binding of small *cis*-diol molecules in the nanoscale pores of DFNS. The boronic acid modified DFNS with the polymer intermediate exhibits higher binding capacity for Alizarin Red S and nicotinamide adenine dinucleotide than the polymer-free DFNS. The two types of boronic acid modified DFNS can bind small *cis*-diol molecules in the presence of large glycoproteins, due in large part to the effect of size exclusion provided by the nanochannels in the DFNS.

## Introduction

In the last decades, mesoporous silica has been extensively studied in materials science for the development of new technologies.^1,2^ Due to their unique properties such as high stability, good biocompatibility, and ease of surface modification, silica nanoparticles are outstanding building blocks in a wide range of applications.^3^ Especially, the large surface area and well-defined pore structure are highly attractive for many practical applications.^4,5^ Kresge and co-workers created Mobil Composite Material (MCM-41), which has ordered hexagonal mesopores.^6^ Stucky and co-workers synthesized silica containing tunable and large mesopores, the material named SBA-15 (Santa Barbara, CA, USA).^7^ A more recent discovery is the dendritic fibrous nanosilica (DFNS) made by Polshettiwar and co-workers. This material has a unique three-dimensional structure in which the hierarchical pores are defined by the numerous center-radial nanochannels.^8^ The highly accessible internal space makes DFNS an ideal platform material to act as a carrier for catalysts, drugs, affinity ligands, and molecular probes.^9^ The large surface of DFNS is helpful to increasing the number of immobilized functional groups and the capacity of the functionalized material. In comparison with conventional mesoporous silica, DFNS has hierarchical porosity in the nanometer range. Its radial-oriented open pore channels allow much faster mass transfer for molecular binding.^10,11^ The well-defined pore volume of DFNS provides a unique size selectivity, because the nanoscale pores are easily entered by small molecules but are not accessible for large molecules.^12^

Chemical conjugation in DFNS allows affinity ligands to be immobilized with a controlled molecular orientation, an important aspect that affects subsequent molecular recognition and separation. For DFNS, ligand immobilization is challenging due to the limited molecular motion in restricted nanoscale pores. The conjugation chemistry therefore must have a very high efficiency. Besides direct fixation on the wall of nanoscale pores, the number of affinity ligands may be increased by immobilization through an intermediate polymer chain, thereby increasing the capacity of the modified nanomaterial. Zhao and co-workers used boronic acid ligand to modify mesoporous silica coating on magnetic microspheres through an amidation reaction, and they used the material to remove endotoxin by magnetic separation.^13^ Luo and co-workers modified the orifice rims of dendritic mesoporous silica nanoparticles with nitrophenyl benzyl carbonate groups, and then they introduced disulfide-linked azido groups on the surface of the inner channel. A final click reaction was used to immobilize polyamidoamine dendrimers into the drug-delivery vehicles.^14^ Using in situ polymerization inside dendrimer-like silica nanoparticles, Liu and co-workers confined polypyrrole in the nanochannels of the silica nanoparticles. A subsequent modification with polyethylene glycol improved the biocompatibility of the nanomaterial.^15^ Yan and co-workers grafted poly(2-(dimethylamino)ethyl methacrylate) on silica nanoparticles via atom transfer radical polymerization (ATRP). The obtained nanoparticles were used to coat an open-tubular capillary to prepare a reversed phase electrochromatography column.^16^

Copper-catalyzed 1,3-dipolar cycloaddition between azide and alkyne is one of the most useful click reactions for modification of solid surfaces because of its very mild reaction conditions,^17,18^ broad functional group tolerance, and high efficiency.^19,20^ To introduce polymer brushes into the nanochannel of DFNS, surface-initiated polymerization techniques such as ATRP are of practical interest because of its capability to control polymer structure and molecular weight. Polymer brushes can be grafted onto the surface conveniently by growing polymer chains from an immobilized ATRP initiator. New functional groups can also be added along the polymer chains by postpolymerization modification.^21^

In this work we investigated the use of Cu(I)-catalyzed alkyne–azide cycloaddition (CuAAC) click reaction and a general-purpose polymer intermediate to immobilize affinity ligands in the nanoscale pores of DFNS. Two types of boronate affinity materials were prepared by direct click conjugation of a phenylboronic acid on silica and conjugation through a flexible, temperature-responsive polymer chain. The obtained nanocomposite materials were evaluated by studying the molecular recognition characteristics for *cis*-diols using Alizarin Red S (ARS) and nicotinamide adenine dinucleotide (NADH) as models. The impact of the intermediate polymer and its temperature-modulated phase variation on target binding were also investigated. The targeted applications of the nanocomposite materials include removal of environmental pollutants, separation of small *cis*-diols from biological samples, and controlled delivery of therapeutic molecules.

## Experimental Section

### Materials

The following chemicals were purchased from Sigma-Aldrich: ammonia solution (NH_4_OH, 25%), ethyl ether (≥99%), hexadecyl trimethylammonium bromide (CTAB, ≥98%), tetraethylorthosilicate (TEOS, ≥99.99%), (3-aminopropyl)-triethoxysilane (APTES, ≥98%), bromoacetyl bromide (≥98%), triethylamine (≥99%), 2-bromoisobutyryl bromide (BIBB, 98%), *N*-isopropylacrylamide (NIPAm, ≥99%), glycidyl methacrylate (GMA, ≥97%), tris(2-dimethylaminoethyl)amine (Me_6_TREN, 96%), copper(I) bromide (99.99%), copper(II) bromide (99%), Alizarin Red S (ARS, grade certified by the Biological Stain Commission), copper(II) sulfate (≥99%), sodium ascorbate, sodium azide (≥99.5%), bovine serum albumin (BSA, ≥98%), ovalbumin from chicken egg white (OVA, ≥98%), methanol (≥99.9%), tetrahydrofuran (THF, ≥98%), isopropanol (99.5%), *N*,*N*-dimethylformamide (DMF, 99.8%), β-nicotinamide adenine dinucleotide, reduced disodium salt hydrate (NADH, ≥97%), acetic acid (≥99%), hydrochloric acid (37%), and lactate dehydrogenase (LDH) from bovine heart (type III, crystalline suspended in ammonium sulfate solution). Ethanol (99.5%) was purchased from Solveco. Prior to use, CuBr was stirred in acetic acid for 24 h, collected by centrifugation, washed with water and methanol, and dried in a vacuum desiccator. 3-(Prop-2-ynyloxycarbonylamino)phenylboronic acid (PCAPBA) was synthesized by use of a literature method.^22^

### Preparation of Amino-Functionalized Dendritic Fibrous Silica (DFNS-NH_2_)

Amino-functionalized dendritic fibrous nanosilica (DFNS-NH_2_) was synthesized with a sol–gel reaction in ethyl ether reported by Du et al.^10^ with slight modification. Typically, 70 mL of water, 1 mL of aqueous ammonia (NH_4_OH, 25%), 20 mL of ethyl ether, and 10 mL of ethanol were added to a 100 mL round-bottom flask and agitated with magnetic stirring vigorously at room temperature for 30 min. CTAB (500 mg) was dissolved into the mixture. After 30 min, a mixture of 2.5 mL of TEOS and 0.1 mL of APTES was added quickly into the above-mentioned mixture. The reaction mixture was stirred vigorously at ambient temperature for 4 h. Next, 1 mL of HCl (37%) was added to quench the reaction. The nanoparticles were isolated by centrifugation, washed with water and ethanol three times, and resuspended in 120 mL of ethanol by sonication, followed by addition of 15 mL of HCl (37%). The mixture was stirred at 70 °C for 24 h, and then the nanoparticles were isolated by centrifugation and washed with ethanol three times to remove the surfactant CTAB from DFNS. The nanoparticles were collected by centrifugation and dried in a vacuum desiccator.

### Preparation of Boronic Acid Modified Dendritic Fibrous Silica (DFNS@BA)

First, DFNS-NH_2_ (100 mg) and THF (15 mL) were added to a 25 mL round-bottom flask. The DFNS-NH_2_ particles were dispersed in THF by sonication, followed by addition of triethylamine (0.5 mL). The suspension was agitated with a magnetic stirrer in an ice–water bath for 15 min. Bromoacetyl bromide (0.5 mL) was added dropwise into the suspension. The reaction mixture was allowed to warm up to room temperature and stirred for 24 h. The nanoparticles were collected by centrifugation, washed three times with water and three times with methanol, and dried in a vacuum desiccator.

Then, the above-obtained particles (100 mg), sodium azide (78 mg), and ammonium chloride (64.5 mg) were dispersed in DMF (7.5 mL). The mixture solution was magnetically stirred at 60 °C for 24 h. After washing with water and methanol, the particles were collected and dried in a vacuum desiccator. The obtained particles were denoted as DFNS@N_3_.

DFNS@N_3_ particles (30 mg) were dispersed in a mixture of methanol (3 mL) and water (3 mL) and then mixed with 1 mL of methanol containing PCAPBA (30 mg). The mixture was sonicated and degassed by application of a vacuum for 15 min. After addition of CuSO_4_ solution (100 mM, 20 μL) and sodium ascorbate solution (1 mM, 200 μL), the reaction mixture was sealed and agitated at 50 °C for 24 h. The nanoparticles were collected and purified following the same procedure as described above.

### Preparation of GMA-NIPAm Copolymer Brushes Grafted on Dendritic Fibrous Silica (DFNS@pco)

DFNS-NH_2_ particles (200 mg) and THF (15 mL) were added to a 25 mL round-bottom flask, and the DFNS particles were dispersed in THF by sonication. After the addition of triethylamine (0.4 mL), the suspension was magnetically stirred in an ice–water bath for 15 min. 2-Bromoisobutyryl bromide (0.31 mL) was added dropwise to the suspension. The reaction mixture was allowed to warm up to room temperature and stirred for 24 h. The nanoparticles were collected by centrifugation, washed three times with water and three times with methanol, and dried in a vacuum desiccator. The initiator-modified DFNS (DFNS@initiator, 50 mg), NIPAm (340 mg), and GMA (210 μL) were dispersed in 8 mL of 2-propanol by sonication, followed by addition of CuBr (7.2 mg, 0.05 mmol) and CuBr_2_ (1.1 mg, 0.005 mmol). The mixture was purged with nitrogen for 15 min, followed by addition of Me_6_TREN (14 μL) to form the CuBr/CuBr_2_/Me_6_TREN ATRP catalyst. After another 15 min of nitrogen bubbling, the reaction mixture was sealed and the polymerization was carried out at 60 °C for 24 h. The nanoparticles were isolated and purified following the same procedure as described above.

### Preparation of Boronic Acid Modified Copolymer Brushes Grafted on Dendritic Fibrous Silica (DFNS@pco@BA)

First, azide groups were introduced into the polymer brushes. The copolymer-modified DFNS (DFNS@pco 100 mg), ammonium chloride (64.5 mg), and sodium azide (78 mg) were dispersed in DMF (7.5 mL). The mixture solution was magnetically stirred at 60 °C for 24 h. After washing with water and methanol, the particles were collected and dried in a vacuum desiccator. The obtained particles were denoted as DFNS@pco@N_3_.

The obtained particles (DFNS@pco@N_3_, 50 mg) dispersed in a mixture of methanol (5 mL) and water (5 mL) were mixed with 2 mL of methanol containing PCAPBA (50 mg). The mixture was deoxygenated by purging with nitrogen gas for 15 min, followed by addition of sodium ascorbate solution (1 mM, 400 μL) and CuSO_4_ solution (100 mM, 40 μL). After another 15 min of nitrogen bubbling, the reaction mixture was sealed and shaken on a rocking table for 24 h at room temperature. The nanoparticles were isolated and purified following the same procedure as described above.

### Confirmation of Boronic Acid Immobilization on DFNS

For proof of successful introduction of boronic acid into the mesoporous silica particles, boronic acid functionalized nanoparticles (1 mg) and 1 mL of 0.1 mM ARS solution (prepared in 20 mM pH 8.5 PBS buffer containing 0.5 M NaCl) were mixed and shaken for 1 h. The fluorescence emission of the nanoparticle suspension was measured by use of a QuantaMaster C-60/2000 spectrofluorometer (Photon Technology International, Lawrenceville, NJ) with an excitation wavelength at 469 nm. For comparison, azide-modified DFNS was used as a control and tested in the same procedure.

### Binding of ARS by DFNS@BA and DFNS@pco@BA

#### Equilibrium ARS Binding Analysis

Boronic acid modified nanoparticles were suspended in phosphate buffer (PBS, 20 mM, pH 8.5, containing 0.5 M NaCl) with a concentration of 2 mg/mL. The particle suspension (0.5 mL) was mixed with 0.5 mL of ARS solution of different concentrations. The samples were gently shaken at 20 °C/40 °C/60 °C at a speed of 200 rpm for 1 h by use of a thermal mixer (Eppendorf, Hamburg, Germany). After this step the samples were immediately centrifuged. The free ARS in the supernatant was measured by use of UV–vis spectrometry (Cary 60 UV–vis, Agilent Technologies, USA). For comparison, the azide-modified particles (DFNS@N_3_) were used as a control and tested in the same procedure.

#### ARS Binding in Different pH Solutions

ARS solution (0.2 mM) was prepared at pH 8.5 (20 mM phosphate buffer, containing 0.5 M NaCl), pH 7.4 (20 mM phosphate buffer, containing 0.5 M NaCl), pH 4 (0.1 M acetate buffer, containing 0.5 M NaCl), and pH 2 (1 M acetic solution, containing 0.5 M NaCl). Boronic acid modified nanoparticles were suspended in corresponding different pH solutions with a concentration of 2 mg/mL. Then, 0.5 mL of boronic acid modified nanoparticles in suspension was mixed with 0.5 mL of ARS solution at different pHs. The samples were gently shaken at 20 °C for 1 h. ARS dissolved in buffer at different pHs has different absorbance peaks in its UV–vis spectrum. The absorbance peak of ARS in phosphate buffer (pH 8.5 and 7.4) is at 515 nm, and in acetate buffer and acetic acid solution it is at 420 nm (Figure S2).

#### ARS Binding in the Presence of Proteins

BSA and OVA (0.5 mL, 0.04 mg/mL) were first separately mixed with 0.5 mL of 120 μM ARS in phosphate buffer (PBS, 20 mM; pH 8.5, containing 0.5 M NaCl). Then 1 mL of the ARS–protein mixture (with a mass ratio of ARS:protein ∼ 1:10) was incubated with 1 mg of DFNS@BA and DFNS@pco@BA for 1 h. The concentration of ARS in supernatant was measured by UV–vis spectrometry at 515 nm.

### Binding of NADH by DFNS@BA and DFNS@pco@BA

The NADH binding procedures were the same as the ARS binding procedures, except that the incubation time was 2 h and the concentration of particles was 2 mg/mL.

### Verification of Protein Exclusion by Monitoring Coenzyme Conversion

LDH suspension (200 μL) was centrifuged for 10 min. The precipitate was dissolved in 200 μL of 20 mM phosphate buffer. The LDH solution was diluted 10 times prior to use.

DFNS@BA particles (2 mg) were dispersed in 20 mM phosphate buffer (0.5 mL, pH 8.5). NADH solution (0.5 mL, 0.2 mM) was added to the particle suspension. The mixture solution was incubated at room temperature for 2 h. After the equilibrium binding, the particles were collected by centrifugation and removal of the supernatant. To the NADH-loaded particles, 960 μL of 20 mM phosphate buffer and 20 μL of LDH solution were added. Upon addition of pyruvate (20 μL, 150 mM), the absorbance at 340 nm of the mixture was recorded by UV–vis spectrometry. Before the kinetic measurement, the UV absorbance was set to zero by using DFNS@BA particles suspended in 20 mM phosphate buffer (2 mg/mL) as reference. For comparison, the azide-modified particles (DFNS@N_3_) were tested following the same procedure.

### Characterization

The functional groups on the modified DFNS were analyzed by a Thermo Fisher Scientific FT-IR instrument (Thermo Fisher Scientific Inc., Waltham, USA). All spectra were collected at room temperature by 16 scans in the 4000–550 cm^–1^ region with a resolution of 4 cm^–1^. Particles were imaged with a scanning electron microscope (JSM-6700F, JEOL, Japan) at 5 kV and a transmission electron microscope (JEM-1400Plus, JEOL, Japan) at 100 kV. The specimens were coated with a gold layer by using Cressington sputter coater 108auto for SEM observation. Before imaging in TEM, the sample (3 μL suspension) was dropped on single slot Pioloform coated copper grid and allowed to dry in air. Nitrogen adsorption–desorption measurements were carried out on an automated surface area analyzer (Model ASAP 2400 of Micrometrics Co., Inc., USA). Specific surface areas and pore size distribution were calculated with the Brunauer–Emmett–Teller (BET) and Barrett–Joyner–Halenda (BJH) methods. Elemental analysis (C, H, N, and B) was performed by Mikroanalytisches Laboratorium Kolbe (Germany). A CHNS Analyzer from Elementar Model Vario Mikro Cube was used for the analysis of C, H, and N elements. Boron was determined by use of a calorimetric method with a Specord 50 Plus spectrometer from Analytik Jena. Thermal gravimetric analysis (TGA) was carried out at the Institute of Polymer Chemistry, Johannes Kepler University Linz, Austria. Samples were heated to 900 °C at a rate of 10 °C/min in synthetic air.

## Results and Discussion

Boronate affinity materials are among the most effective for separation and enrichment of *cis*-diol-containing molecules such as carbohydrates, catechols, nucleosides, glycoproteins, and bacteria.^23,24^ The performances of boronic acid based adsorbents are dependent on both the affinity ligands and the supporting materials.^25^ Nanomaterial-supported boronic acid ligands exhibit several significant advantages including fast adsorption/desorption kinetics, pH-controlled capture/release process, and broad-spectrum affinity.^26^ Because of its unique structural characteristics, DFNS can add one additional selectivity defined by molecular sizes in addition to the well-known boronic acid–*cis*-diol interactions. DFNS-based boronate adsorbents with stimuli-responsive molecular binding properties are promising new materials for a number of biochemical and medical applications.

### Synthesis and Characterization of Nanoparticles

**Scheme 1:**
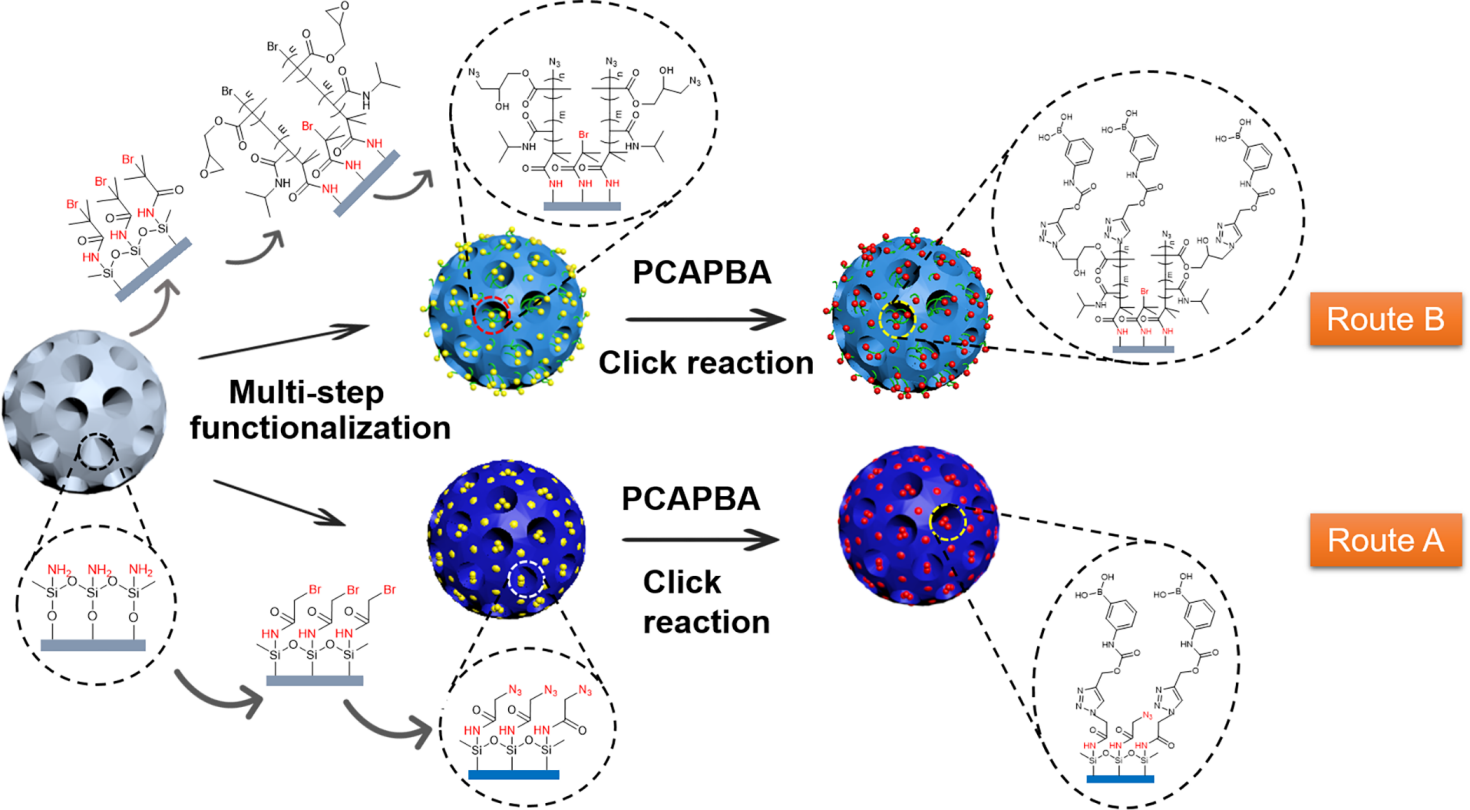
Synthetic Approaches for Preparation of Boronic Acid Functionalized DFNS

Generally, small molecular ligands such as boronic acids can be bonded onto solid supports via direct conjugation or through an intermediate polymer. In this work, we studied the use of CuAAC reaction to introduce a clickable boronic acid into narrow pores of DFNS in two different routes: direct conjugation of alkyne-tagged phenylboronic acid (Scheme 1, route A) and attachment of boronic acid to a thermoresponsive polymer chain grafted inside the narrow pores of DFNS (Scheme 1, route B). The process of material synthesis for DFNS@BA and DFNS@pco@BA particles is illustrated in Scheme 1. DFNS-NH_2_ was synthesized with the use of a literature method.^10^ In the route of attachment of boronic acid to a thermoresponsive polymer chain grafted inside the narrow pores of DFNS (route B), the amino groups on the internal surface are converted into alkyl bromide, with the Br-modified surface leading to the polymer-containing nanoparticles. *N*-Isopropylacrylamide (NIPAm) and glycidyl methacrylate (GMA) were used as monomers to graft a copolymer in the nanochannel of the DFNS by ATRP. GMA was used to introduce the azide group into the copolymer because its epoxy group can undergo ring-opening reaction with sodium azide. The copolymerization with NIPAm was used to make the polymer thermally responsive. Thus, the copolymer has a lower critical solution temperature (LCST) at around 32 °C in aqueous solution. Below the LCST the polymer has a random coil structure, but above the LCST it forms a more collapsed globular structure. Normally, boronic acids require a basic pH (8.5 or higher) to form strong binding with compounds containing *cis*-diol structure.^27^ To overcome this problem, an alkynyl tagged boronic acid (PCAPBA) was linked to the copolymer chain by CuAAC click reaction. As the triazole nitrogen is located in the vicinity of the boron in the phenylboronic acid, the B atom can be kept in a sp^3^ configuration even at neutral pH. Consequently, the triazole-linked boronic acid is able to bind *cis*-diols under neutral pH conditions.^28^ In the route of direct conjugation (route A), the same Br-modified DFNS leading to the polymer-containing nanoparticles is first converted to N_3_-modified DFNS (DFNS@BIBB@N_3_) and then modified with PCAPBA by CuAAC click reaction. However, PCAPBA was found difficult to react with the alkyl azide, as shown in Figure S1, where the azide signal in the FT-IR spectrum did not disappear after the click reaction. This result can be explained by that the two methyl groups adjacent to the azido group inhibit the CuAAC reaction due to steric congestion.^29^ Thus, bromoisobutyryl bromide was changed to bromoacetyl bromide to introduce a more reactive alkyl azide to the DFNS for direct immobilization of PCAPBA.

**Figure 1:**
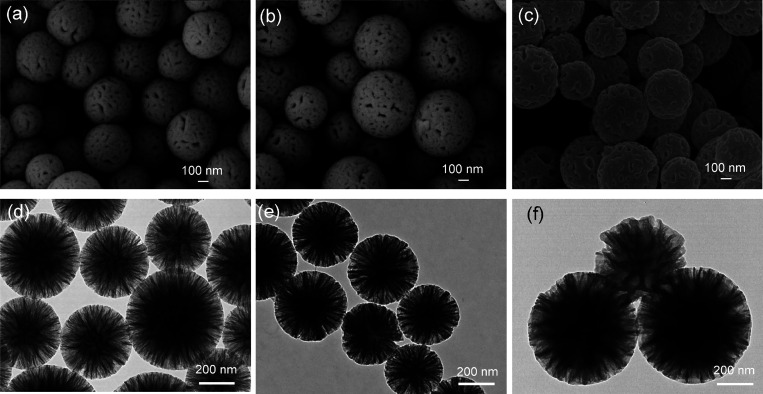
SEM and TEM images of DFNS-NH_2_ (a, d), DFNS@BA (b, e), and DFNS@pco (c, f).

**Figure 2:**
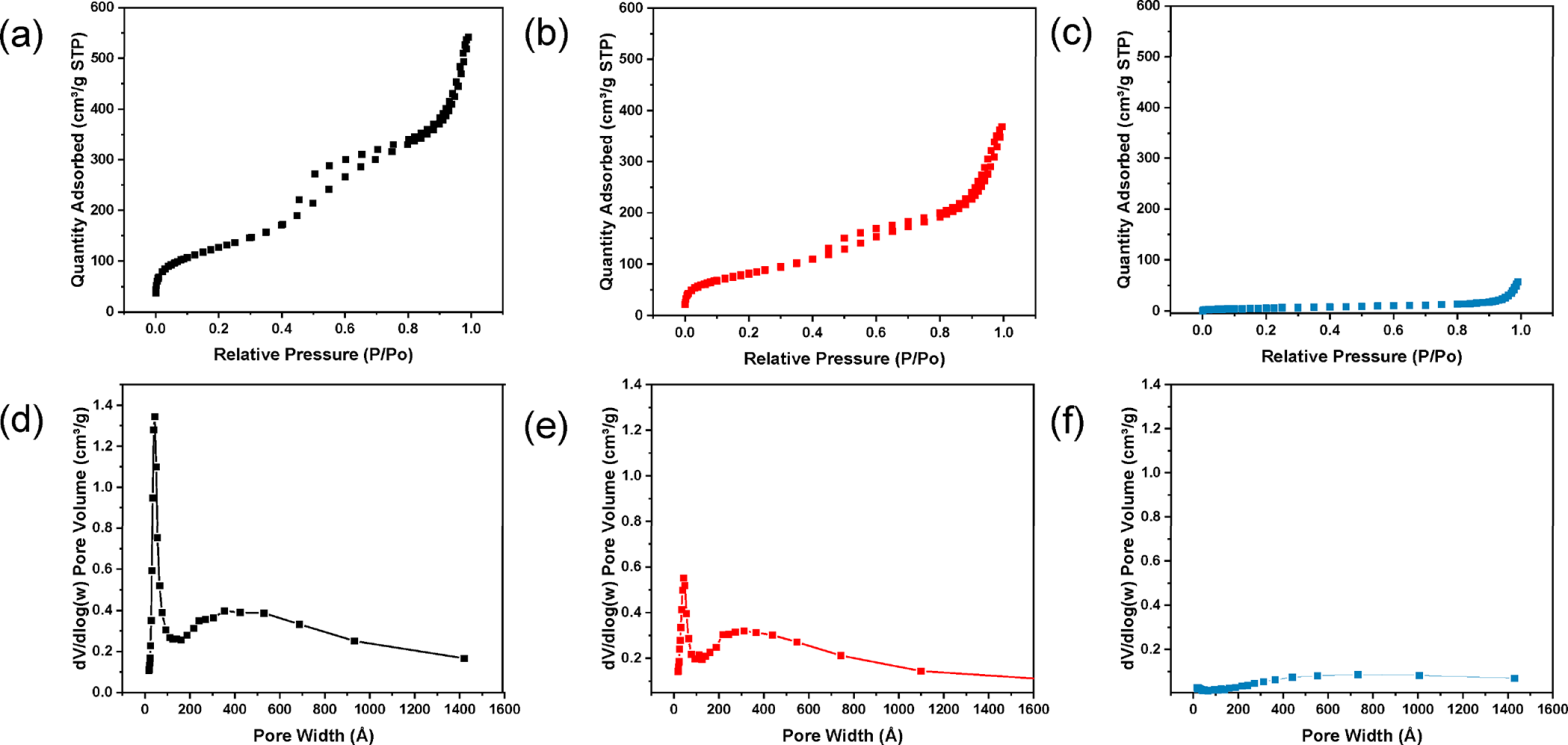
Nitrogen adsorption–desorption isotherms and pore size distributions of DFNS-NH_2_ (a, d), DFNS@BA (b, e), and DFNS@pco (c, f).

Scanning electron microscopy and transmission electron microscopy showed that the DFNS has a uniform and spherical shape with a central-radial pore structure (Figure 1a,d). BET and BJH methods were employed to calculate the surface area and the pore size. The nitrogen adsorption/desorption data in Figure 2a shows a type IV isotherm with a type H4 hysteresis loop.^10^ The mesopores in the DFNS are characterized as slit-shaped that may result from the central-radial channels in the DFNS. The pore size distribution plot shows a sharp and strong peak at 4.25 nm (Figure 2d). The shallow and wide peak between 16 and 80 nm may result from the interparticle space between the aggregated particles. Thus, the BET surface area and pore size of DFNS are 460 m^2^/g and 4.25 nm, respectively. After modification with boronic acid, there is no obvious change in the morphology on the modified particles (DFNS@BA; Figure 1b,e). The surface area decreased slightly to 301 m^2^/g and the pore size remained 4.27 nm (Figure 2b,e). In contrast to the addition of small molecular ligands, the surface-initiated polymerization caused the mesoporous channel to be filled up completely with the polymer chains (Figure 1c,f). The BET surface area of DFNS@pco decreased remarkably to 22.4 m^2^/g, and there are no noticeable mesopores left in the polymer-filled nanoparticles (Figure 2c,f).

**Figure 3:**
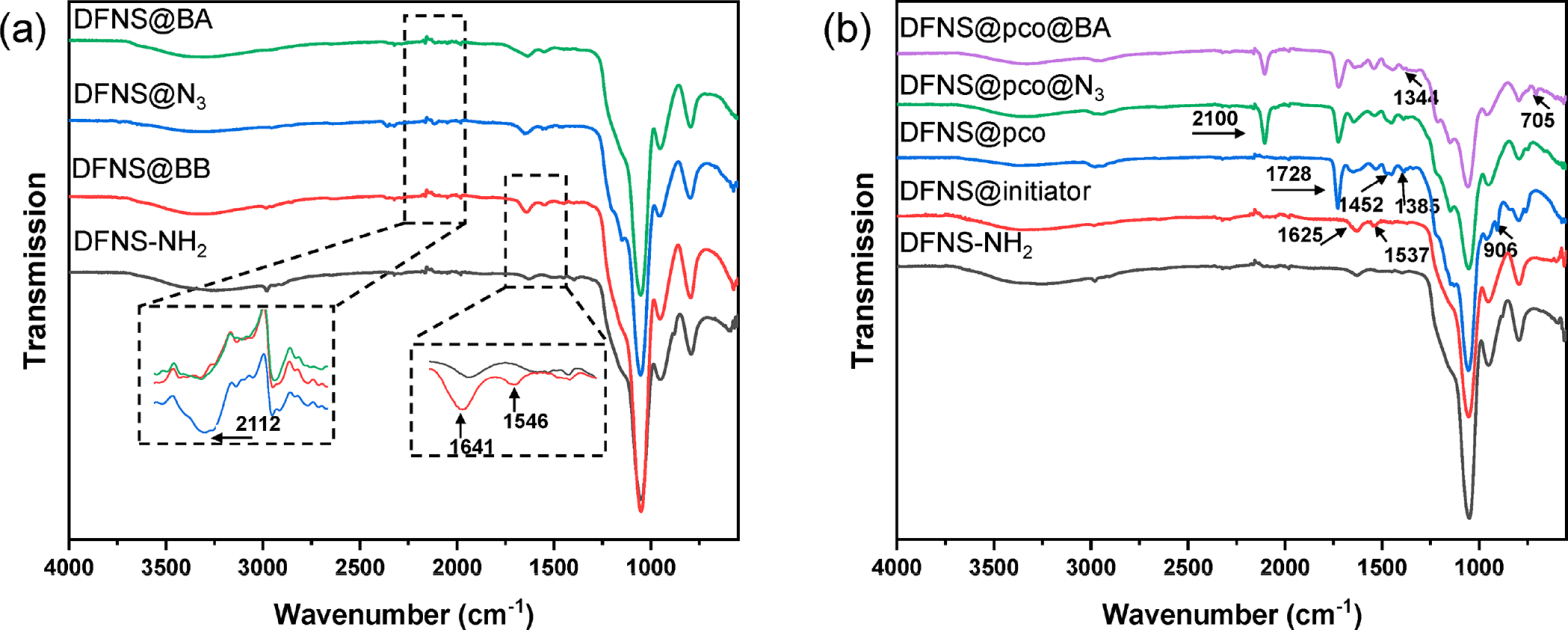
FT-IR spectra of DFNS-NH_2_ particles before and after modification with different small organic molecules (a). FT-IR spectra of DFNS-NH_2_ particles before and after modification with ATRP initiator and with different polymer structures (b).

FT-IR spectroscopy was used to analyze the chemical composition of the nanoparticles obtained after different synthetic steps. As shown in Figure 3, for bare DFNS-NH_2_, IR signals corresponding to Si–O–Si symmetrical stretching vibration (804 cm^–1^), Si–OH bending vibration (955 cm^–1^), and Si–O asymmetric stretching vibration (1080 cm^–1^) were observed. For DFNS@BB, a band was observed at 1641 cm^–1^ due to C=O stretching of the amide I band and another band was observed at 1546 cm^–1^ due to N–H stretching of the amide II band.^28^ After the terminal bromide was turned into an azide group, an azide signal at 2112 cm^–1^ appeared from DFNS@N_3_. After the click reaction, the intensity of this azide signal decreased significantly, which suggests that boronic acid had been linked to DFNS. For DFNS@initiator, its FT-IR spectrum was nearly identical to that of the DFNS modified with bromoacetyl bromide, as the initiator 2-bromoisobutyryl bromide has the same chemical structure as bromoacetyl bromide except for the two methyl groups. After grafting the copolymer brushes, DFNS@pco exhibited a band at 1728 cm^–1^ due to the stretching vibration of the ester carbonyl group and another band at 906 cm^–1^ due to the symmetrical stretching of the epoxy group from the monomer GMA. Furthermore, two bands at 1385 and 1452 cm^–1^ are assigned to the methyl groups of the isopropyl moiety and the methylene scissoring band from the copolymer. For DFNS@pco@N_3_, a strong azide signal at 2100 cm^–1^ can be observed.^21^ For DFNS@pco@BA, part of the azide signal can still be observed. This incomplete conversion of azide into boronic acid may be caused by a steric hindrance during the click reaction. Besides, the two IR bands at 1334 and 705 cm^–1^ are assigned to boron–oxygen bond stretching vibration and carbon–hydrogen bond vibration, respectively.^30^

To further confirm that the boronic acid was successfully introduced into DFNS@BA and DFNS@pco@BA, ARS was used as a fluorogenic reporter to detect boronic acids. As shown in Figure S3, after being treated with ARS, DFNS@BA and DFNS@pco@BA displayed an emission peak near 600 nm that was not observed from DFNS@N_3_ and DFNS@pco@N_3_. This result confirmed the presence of boronic acid in the two types of boronic acid modified nanoparticles. Besides, the intensity of fluorescence from DFNS@pco@BA was higher than that from DFNS@BA, which confirmed the amount of boronic acid modified on DFNS@pco@BA was higher than that on DFNS@BA.

Elemental analysis and thermogravimetric analysis were used to assess the composition and calculate the organic content of the DFNS. The experimental results are summarized in Table 1 and Figure S4. The N element in DFNS originated from APTES (Table 1, entry 1). On the basis of the N content, the amino groups present in the bare DFNS-NH_2_ were determined to be 0.44 mmol/g of DFNS. The N and B contents in DFNS@BA further increased after multistep modifications on the DFNS. After grafting the copolymer and introducing the boronic acid, the contents of C, H, N, and B increased dramatically. From the TGA data, the residual weight after the thermodecomposition can be used to calculate the content of the inorganic silica material. Based on the content of B from the elemental analysis and inorganic silica from TGA, the amounts of boronic acid ligand in DFNS@BA and DFNS@pco@BA were 0.08 and 0.68 mmol of boronic acid/g of silica, respectively. The density of boronic acid in DFNS@pco@BA is therefore much higher than that in DFNS@BA.

**Table 1:** Results of Elemental Analysis of Nanoparticles

entry	sample name	% C	% H	% N	% B
1	DFNS	7.09	3.31	0.56	<0.01
2	DFNS@BA	6.99	2.75	1.93	0.07
3	DFNS@pco@BA	34.56	5.15	8.80	0.23

The TGA curve for DFNS@pco in Figure S4 shows a main decomposition in the temperature range 200–400 °C, which can be attributed to the thermal decomposition of the copolymer grafted on the DFNS.^21^ After the temperature reached 900 °C, the remaining weight was 38.87%. The weight loss below 200 °C (∼4.12%) is caused by the evaporation of residual solvent and water. Thus, the copolymer content in DFNS@pco is calculated to be 57.11%. Consequently, the amount of poly(NIPAm-*co*-GMA) grafted on the mesoporous silica is approximately 1.47 g/g of DFNS.

### Evaluation of *cis*-Diol Binding Using ARS as Model

ARS is a historically important dye.^31^ It contains a *cis*-diol structure and can be used as a molecular probe to bind phenyboronic acid in a broad pH range. Besides, the removal of ARS from wastewater is an important environmental issue.^32^ In this work, ARS with a molecular weight of 360.3 g/mol was used as a low-molecular-weight *cis*-diol representative to study the molecular binding properties of the mesoporous silica nanoparticles.

#### Effect of pH on ARS Binding

**Figure 4:**
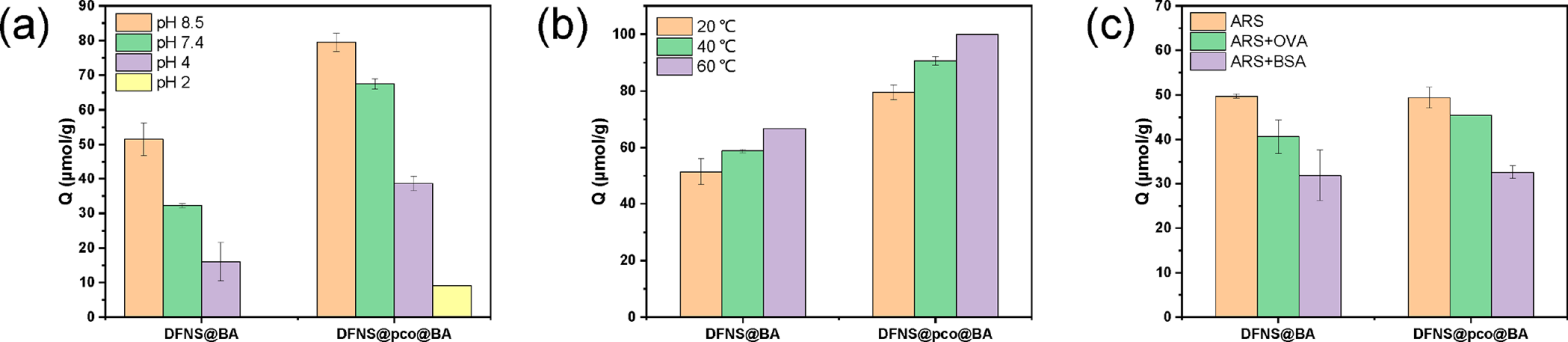
(a) ARS binding on boronic acid modified DFNS measured at 20 °C and different pH values. (b) ARS binding on boronic acid modified DFNS measured at pH 8.5 and different temperatures. (c) ARS binding on boronic acid modified DFNS measured at 20 °C and pH 8.5 in the presence of proteins. In (a) and (b), the initial concentration of ARS was 100 μM. In (c), the initial concentration of ARS was 60 μM. The concentration of the composite particles was 1 mg/mL.

The binding of ARS to the DFNS particles under different conditions is shown in Figure 4. As shown in Figure 4a, both DFNS@BA and DFNS@pco@BA particles displayed clear pH-dependent binding for ARS. The ARS binding decreased significantly when the buffer pH changed from 8.5 to 4. In acetic acid solution at pH 2.0, the ARS binding decreased further. Almost no ARS binding was observed on DFNS@BA, and only a small amount of ARS bound to DFNS@pco@BA. The pH-dependent ARS binding is due to the change of structure of the appended phenylboronic acid. Normally, a pH higher than 8.5 can enhance *cis*-diol binding to boronic acid. In the case of ARS, it is known that ARS binding with soluble boronic acid is not sensitive to pH variation between pH 6 and 9.^33^ Considering that molecular recognition in natural environment mostly takes place at close to neutral pH, we did not test ARS binding at pHs higher than 8.5 in this work.

#### Effect of Temperature on ARS Binding

As can be clearly seen in Figure 4b, the capacities of DFNS@BA and DFNS@pco@BA for ARS binding became higher when the temperature was increased. For DFNS@BA, it may be that the higher temperature accelerated the collision frequency of ARS with the boronic acid modified on DFNS. For DFNS@pco@BA, except for more frequent molecular collisions, the boronic acid ligands may become more easily accessed at higher temperature, because the immobilized polymer chains became more hydrophobic and collapsed above the 32 °C LCST. Consequently, when the temperature was increased, more space became accessible in the nanochannels after the polymer chains collapsed, allowing ARS to enter the nanochannels more easily to bind to the boronic acid.

#### Isotherm of ARS Binding

**Figure 5:**
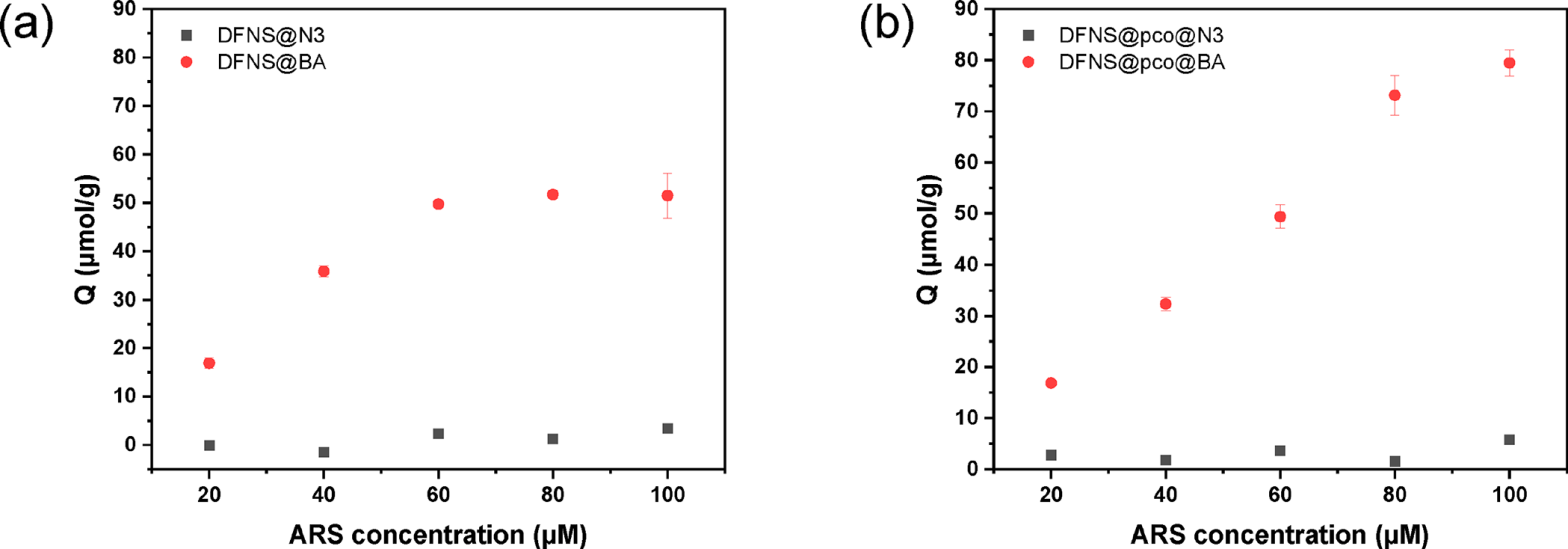
(a) ARS binding on small molecule modified DFNS (DFNS@N_3_ and DFNS@BA) at 20 °C and pH 8.5. (b) ARS binding on polymer-containing DFNS (DFNS@pco@N_3_ and DFNS@pco@BA) at 20 °C and pH 8.5. The particle concentration was 1 mg/mL.

The capacity and strength of *cis*-diol binding of the DFNS particles were studied in phosphate buffer (20 mM, pH 8.5, containing 0.5 M NaCl). ARS was incubated with DFNS@BA and DFNS@pco@BA until equilibrium to establish the binding curve. The addition of NaCl was to reduce nonspecific adsorption caused by electrostatic interactions between ARS and the boronic acid groups. For comparison, the particles without boronic acid (DFNS@N_3_ and DFNS@pco@N_3_) were used as controls in the same binding experiments. As shown in Figure 5, the binding capacities of DFNS@BA and DFNS@pco@BA for ARS are 51.5 and 79.5 μmol/g, respectively. The polymer-containing nanoparticles have a much higher capacity than the polymer-free particles. By contrast, without the boronic acid ligand, the binding capacities of DFNS@N_3_ and DFNS@pco@N_3_ are significantly lower (3.44 and 5.79 μmol/g). The higher *cis*-diol binding capacity of DFNS@pco@BA is attributed to the higher density of boronic acid ligands immobilized on the flexible polymer chains inside the DFNS.

### Binding of ARS in the Presence of Proteins

As the average pore size of the DFNS was about 4.25 nm and the majority of boronic acid was located inside the nanochannels, large molecules such as proteins will not be able to enter the nanochannels to react with the boronic acid, while small *cis*-diols can more easily bind to the internal boronic acid. In order to evaluate the capabilities of DFNS@BA and DFNS@pco@BA to capture small *cis*-diol molecules regardless of competing glycoproteins, further ARS binding experiments were performed in the presence of ovalbumin (OVA) and bovine serum albumin (BSA). BSA is a nonglycoprotein with a molecular weight of 66 kDa and a size of 12 × 4 × 4 nm, and OVA is a glycoprotein with a molecular weight of 42.7 kDa and a size of 7 × 4.5 × 5 nm.^34^ BSA and OVA were first separately mixed with ARS with a mass ratio of ∼10:1; then the ARS–protein mixtures were incubated with DFNS@BA and DFNS@pco@BA for 1 h. The concentration of ARS in supernatant was measured by UV–vis spectrometry at 515 nm. Note that the IR absorption band of ARS was not affected by the proteins (Figure S5). As shown in Figure 4c, in the pure ARS solution (60 μM), the ARS binding on DFNS@BA and that on DFNS@pco@BA were 49.7 and 49.4 μmol/g. When 10-fold OVA was added, the ARS binding to DFNS@BA and that to DFNS@pco@BA decreased only slightly to 40.6 and 45.5 μmol/g, respectively, which are more than 80% of the original binding. Besides, considering the deviation of the measured values, there was no big difference after addition of the glycoprotein OVA. Compared to OVA, addition of BSA in the binding solvent caused a larger reduction of ARS binding to DFNS@BA and DFNS@pco@BA, with the ARS binding becoming 31.9 and 32.7 μmol/g, respectively. The reduction of ARS binding to the nanoparticles can be explained as a result of the association of ARS with BSA,^35^ which formed a large ARS–BSA complex not able to enter the nanochannels in the DFNS particles.

### Evaluation of NADH Binding

**Figure 6:**
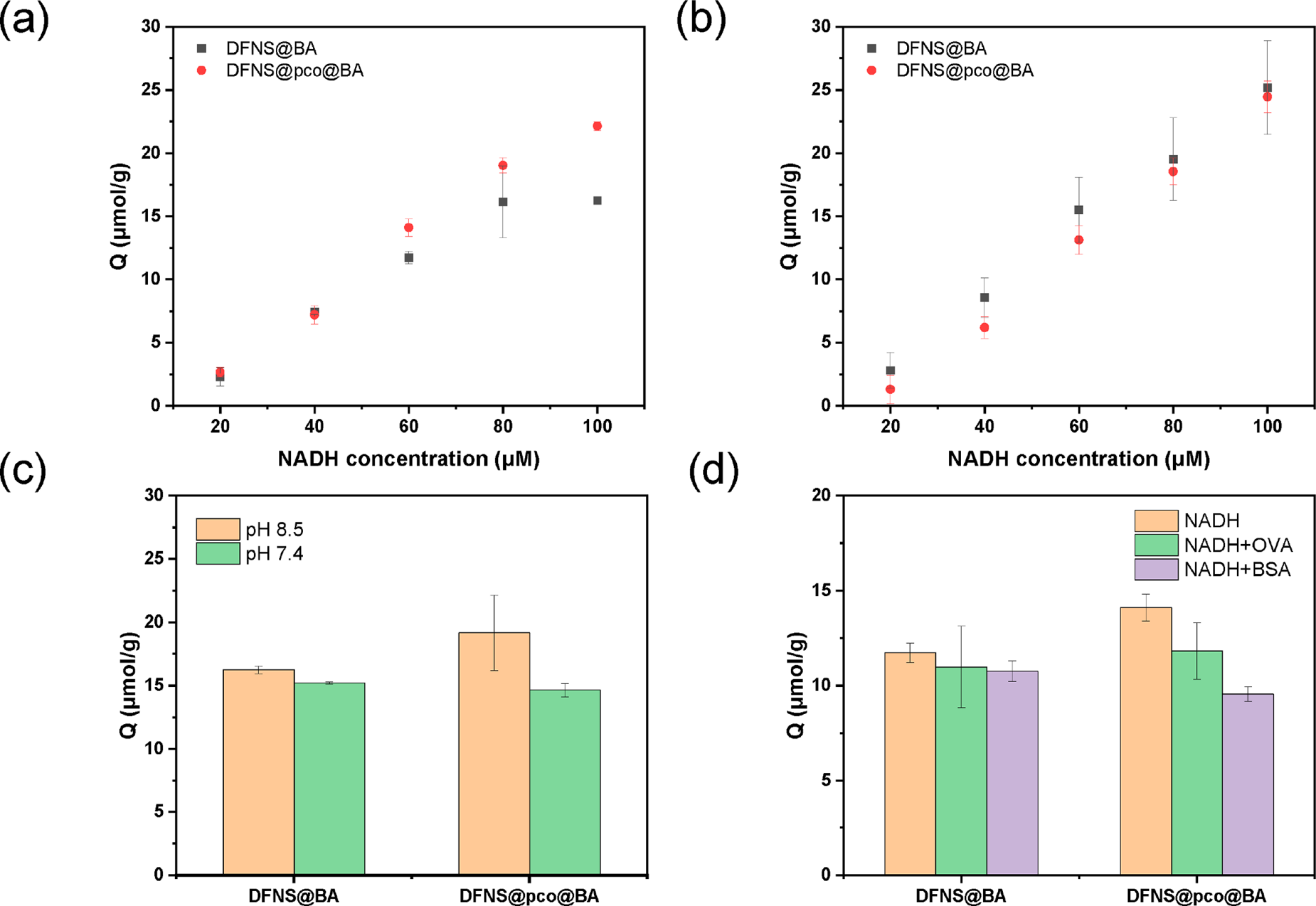
(a) NADH binding on boronic acid modified DFNS at 20 °C and pH 8.5. (b) NADH binding on boronic acid modified DFNS at 40 °C and pH 8.5. (c) NADH binding on boronic acid modified DFNS at 20 °C and different pH values. The initial concentration of NADH was 100 μM. (d) NADH binding on boronic acid modified DFNS at 20 °C and pH 8.5 in the presence of proteins. The initial concentration of NADH was 60 μM. The particle concentration was 1 mg/mL.

DFNS@BA and DFNS@pco@BA particles were also tested for binding to a larger *cis*-diol, NADH with a molecular weight of 663.4 g/mol. NADH is an important coenzyme used by many dehydrogenases.^36^ The binding curves of NADH measured on DFNS@BA and DFNS@pco@BA at 20 and 40 °C are shown in Figure 6a,b. At 20 °C, the NADH binding on DFNS@pco@BA and that on DFNS@BA were 22.1 and 16.3 μmol/g, with DFNS@pco@BA showing a higher capacity. At 40 °C, the NADH binding on DFNS@pco@BA and that on DFNS@BA were 24.5 and 25.2 μmol/g. The NADH binding on both particles increased at higher temperature; however, the temperature effect on NADH binding with DFNS@pco@BA was smaller than on that with DFNS@BA. Presumably, due to its larger molecular size, it is more difficult for NADH to enter the narrow pores in DFNS@pco@BA than for ARS even when the temperature is above the LCST. Without the boronic acid ligand, almost no NADH binding was observed on DFNS@N_3_ and DFNS@pco@N_3_ particles (Figure S6).

The NADH binding offered by the two kinds of particles was also investigated at physiological condition (pH 7.4). As shown in Figure 6c, at pH 7.4, the binding of NADH to DFNS@BA and that to DFNS@pco@BA were only slightly lower than at pH 8.5. Thus, the two types of particles are suitable for separation of NADH from biological samples.

DFNS@BA and DFNS@pco@BA were tested for selective binding of NADH in the presence of proteins. NADH (60 μM, ∼0.02 mg/mL) and the proteins (OVA or BSA, 0.2 mg/mL) dissolved in phosphate buffer (20 mM, pH 8.5, containing 0.5 M NaCl) were incubated with DFNS@BA and DFNS@pco@BA particles. As shown in Figure 6d, the NADH binding on DFNS@BA and that on DFNS@pco@BA were 11.7 and 14.5 μmol/g in the absence of the proteins. The binding capacities of DFNS@BA and DFNS@pco@BA for NADH became 10.8 and 10.9 μmol/g after 10-fold OVA was added. These values are still more than 80% of the original binding capacity for NADH. For DFNS@pco@BA particles, addition of BSA caused the NADH binding capacity to reduce to 9.6 μmol/g. The reduction of NADH binding to DFNS@pco@BA particles was caused by the NADH–BSA complexation,^37^ similar to the effect of BSA on ARS binding. For DFNS@BA particles, no obvious reduction of NADH binding was observed after addition of BSA. Because DFNS@BA has more accessible space in the nanochannels for NADH than DFNS@pco@BA has, the coenzyme can bind more easily to the polymer-free nanoparticles via the boronate ester bond.

### Verification of Protein Exclusion

**Figure 7:**
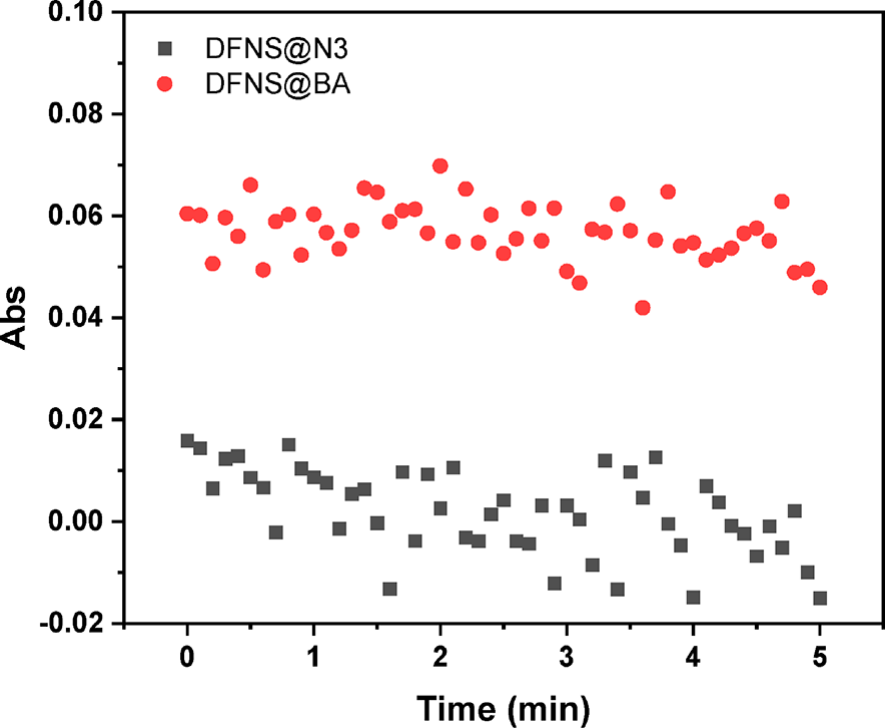
Variation of UV absorbance of NADH-loaded particles monitored at 340 nm. The measurement started immediately after addition of pyruvate and LDH. Background absorbance from the particles has been removed.

NADH can be oxidized to NAD^+^ during the conversion of pyruvate to lactate catalyzed by LDH. The UV absorbance of NADH at 340 nm can be used to monitor the concentration of the coenzyme. Figure 7 shows the change of UV absorbance of the NADH-loaded particles after addition of pyruvate and LDH. The absorbance of DFNS@BA particles (*A*_340_ = 0.06) was higher than that of the DFNS@N_3_ particles (*A*_340_ = 0.015) at the very beginning, because more NADH was loaded to DFNS@BA than to DFNS@N_3_ particles. This result also suggests that the DFNS@BA particles have higher binding capacity for NADH due to the immobilized boronic acid (Figure S6). As seen in Figure 7, after addition of pyruvate and LDH to the NADH-loaded particles, the absorbance of DFNS@BA and that of DFNS@N_3_ at 340 nm exhibited no obvious change over a period of 5 min, indicating that no NADH was converted to NAD^+^. Because the boronic acid ligand is located inside the narrow pores, DFNS@BA particles can act as an effective nanocarrier for NADH and protect it from being oxidized by the large redox enzyme LDH that has a molecular size of 6 × 8.6 × 13.6 nm.^38^

The present work has focused on boronic acid functionalized DFNS for binding small *cis*-diol molecules. The nanoscale pores in the amino-functionalized DFNS-NH_2_ particles acted as a size-selective gate to allow only low-molecular-weight *cis*-diols to reach the boronic acid ligand. By using DFNS particles with different fiber density or defective DFNS as scaffolds,^39,40^ we expect it will be possible to fine-tune the size selectivity and to increase the binding capacity of new affinity nanomaterials.

## Conclusion

Two types of boronic acid functionalized DFNSs, DFNS@pco@BA and DFNS@BA, have been synthesized successfully to enable effective binding of small *cis*-diol molecules. The large amount of immobilized boronic acid can provide more affinity sites for *cis*-diol enrichment, and the well-defined narrow pores of the supporting silica provide high selectivity of affinity binding toward low-molecular-weight *cis*-diols. Besides, the immobilized boronic acid ligand exhibits effective binding for *cis*-diols under neutral pH conditions, and the temperature-responsive copolymer added in the nanochannels makes it possible to control the accessibility of the immobilized affinity ligand. This work opens up new possibilities of introducing molecular recognition ligands into DFNS, with the nanocomposite products having a wide range of applications including drug delivery, biosensing, and catalysis.

## Supporting Information Available

FT-IR spectra of DFNS@BIBB and DFNS@BIBB@BA particles; UV spectra of ARS solutions at different pH values; TGA analysis; fluorescence emission of boronic acid modified DFNS after mix with ARS; UV spectra of mixture of ARS with BSA or OVA; NADH binding measured with DFNS@N_3_ and DFNS@pco@BA particles (PDF)
