# Supplementary material for: Boronic Acid Functionalized Nanosilica for Binding Guest Molecules
Source: ACS Appl Nano Mater. 2021 Feb 19;4(3):2866–75. doi: 10.1021/acsanm.1c00005 (PMC8029584; doi:10.1021/acsanm.1c00005)
Supplement: Supplementary file 1 — an1c00005_si_001.pdf [file an1c00005_si_001.pdf]

Supporting Information

Boronic Acid-Functionalized Nanosilica for Binding Guest Molecules

*Xiaoting Xue, Haiyue Gong, Hongwei Zheng, and Lei Ye**

Division of Pure and Applied Biochemistry, Department of Chemistry, Lund University, Box 124, 22100 Lund, Sweden

Corresponding author: Lei Ye, Email: lei.ye@tbiokem.lth.se
Tel.: +46 46 2229560

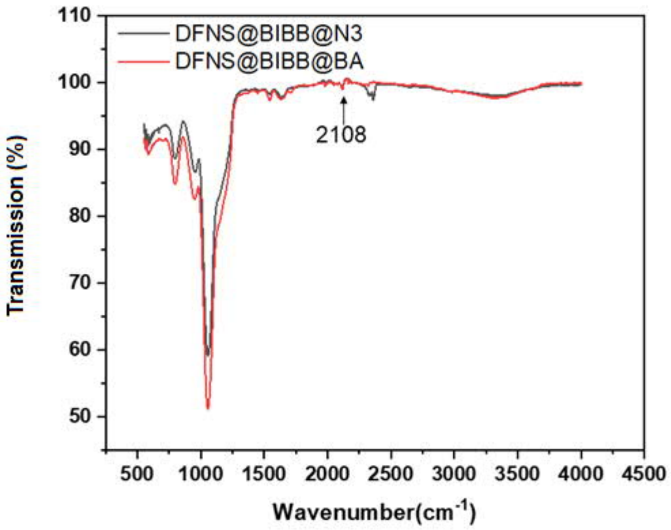

Figure S1. FT-IR spectra of DFNS@BIBB@N_3 and DFNS@BIBB@BA particles. DFNS@BIBB@BA was obtained by reacting DFNS@BIBB@N_3 with PCAPBA.

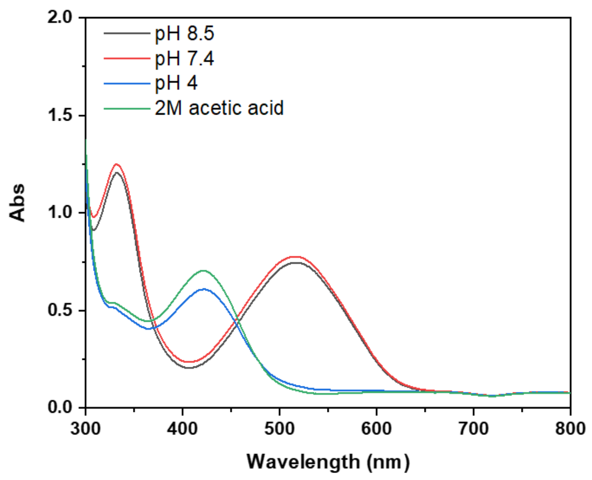

Figure S2. UV-vis spectra of ARS in 20 mM phosphate buffer at pH 7.4 and 8.5, measured in 0.2 M acetate buffer at pH 4 and 2 M acetic acid solution (pH 2.5).

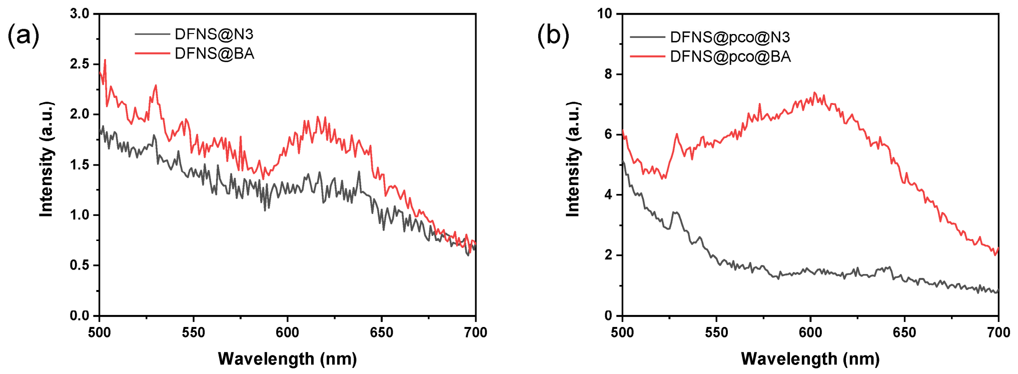

Figure S3. Fluorescence emission of boronic acid-modified DFNS (a) and boronic acid-modified DFNS containing the copolymer (b) after mixing with ARS.

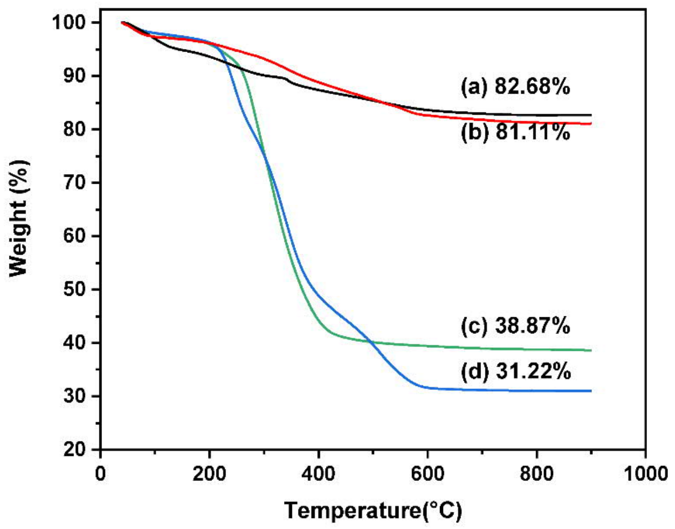

Figure S4. TGA analysis of (a) DFNS, (b) DFNS@BA, (c) DFNS@pco and (d) DFNS@pco@BA particles.

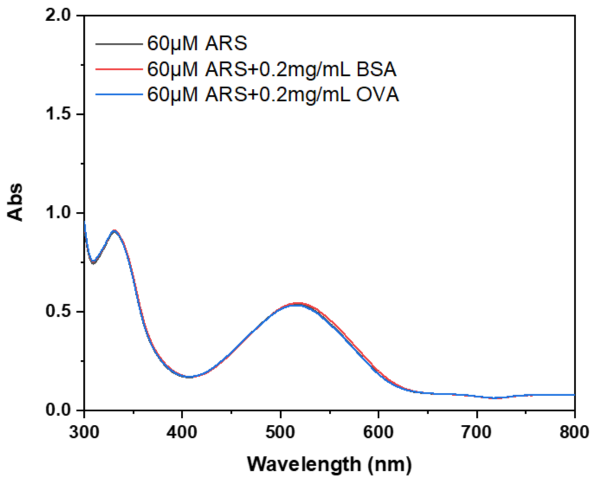

Figure S5. UV-vis spectra of ARS solution before and after addition of BSA and OVA.

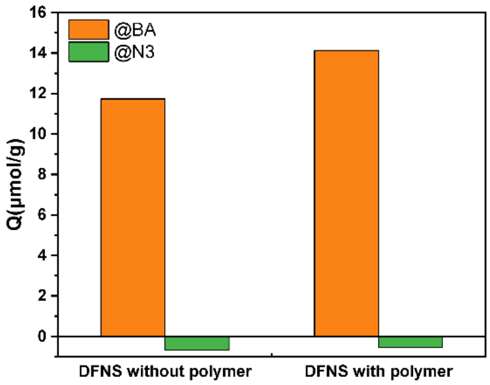

Figure S6. NADH binding measured with DFNS@N_3 and DFNS@pco@BA particles.
